# Slow ring flips in aromatic cluster of GB1 studied by aromatic ^13^C relaxation dispersion methods

**DOI:** 10.1007/s10858-020-00303-3

**Published:** 2020-02-03

**Authors:** Matthias Dreydoppel, Heiner N. Raum, Ulrich Weininger

**Affiliations:** Institute of Physics, Biophysics, Martin-Luther-University Halle-Wittenberg, 06120 Halle (Saale), Germany

**Keywords:** Aromatic interaction, NMR spectroscopy, Protein dynamics, Protein breathing, Protein stability

## Abstract

Ring flips of phenylalanine and tyrosine are a hallmark of protein dynamics. They report on transient breathing motions of proteins. In addition, flip rates also depend on stabilizing interactions in the ground state, like aromatic stacking or cation–π interaction. So far, experimental studies of ring flips have almost exclusively been performed on aromatic rings without stabilizing interactions. Here we investigate ring flip dynamics of Phe and Tyr in the aromatic cluster in GB1. We found that all four residues of the cluster, Y3, F30, Y45 and F52, display slow ring flips. Interestingly, F52, the central residue of the cluster, which makes aromatic contacts with all three others, is flipping significantly faster, while the other rings are flipping with the same rates within margin of error. Determined activation enthalpies and activation volumes of these processes are in the same range of other reported ring flips of single aromatic rings. There is no correlation of the number of aromatic stacking interactions to the activation enthalpy, and no correlation of the ring’s extent of burying to the activation volume. Because of these findings, we speculate that F52 is undergoing concerted ring flips with each of the other rings.

**Electronic supplementary material:**

The online version of this article (10.1007/s10858-020-00303-3) contains supplementary material, which is available to authorized users.

## Introduction

Aromatic residues are overrepresented in protein binding interfaces where they contribute to a significant part of the binding free energy. They also contribute to a significant part (roughly 25% of the volume in average) of the hydrophobic core where they stabilize proteins in two ways. Firstly, they are hydrophobic (especially Trp and Phe) and contribute to the so called hydrophobic effect, where hydrophobic side chains are excluded from the solvent (water) (Pace et al. 2014; Rose and Wolfenden 1993). Secondly, due to their quadrupolar electrostatic character, they can be engaged in specific aromatic-aromatic pair interactions (Burley and Petsko 1985, 1989) and interact with cations (Mahadevi and Sastry 2013) or sulfur (Valley et al. 2012).

Additionally, many Phe and Tyr residues undergo frequent 180° rotations ("ring flips") of the χ_2_ dihedral angle (around the imaginary C_β_–C_γ_–C_ζ_ axis) (Campbell et al. 1975; Hattori et al. 2004; Hull and Sykes 1975; Wagner et al. 1976, 1987; Weininger et al. 2013, 2014b; Wüthrich and Wagner 1975; Yang et al. 2015). The requirement for a ring flip to occur is that the surrounding undergoes concerted "breathing" motions with relatively large activation volumes (Hattori et al. 2004; Li et al. 1999; Wagner 1980). Thus, aromatic side chains are the perfect probe for such transient dynamic processes in proteins. Additionally, a ring flip directly reports on the energy difference between the ground state and the transition state (90° tilted ring). Because of the quadrupolar electrostatic nature, interactions that are stabilizing the ground state are destabilizing the transition state and thus are leading to slower ring flips. Comparing ring flips for aromatic residues involved in different interactions should therefore provide an experimental measure of the energy of these interactions.

Experimental measurements of ring flips, however, have been limited so far to a handful of cases since their discovery in the 1970s (Campbell et al. 1975; Hull and Sykes 1975; Wagner et al. 1976). Recently, new cases have been reported (Weininger et al. 2014b; Yang et al. 2015) enabled by methodological advances in site-selective ^13^C labeling (Lundström et al. 2007; Teilum et al. 2006), aromatic ^13^C relaxation dispersion experiments (Weininger et al. 2012, 2014a) and the understanding of strong ^1^H–^1^H couplings (Weininger et al. 2013). Additionally, ring flips can be studied by long scale MD simulations (Shaw et al. 2010) and extremely fast ring flips are shown to affect order parameters (Kasinath et al. 2015). So far, ring flips in all but one system (Nall and Zuniga 1990) are of aromatic residues without specific interactions, like aromatic-aromatic pair interactions (Burley and Petsko 1985, 1989) and interactions with cations (Mahadevi and Sastry 2013) or sulfur (Valley et al. 2012). They all show a similar activation enthalpy of 83–97 kJ mol^−1^ (Hattori et al. 2004; Weininger et al. 2014b), while for Iso-2-cytochrome c higher activation enthalpies of 117–150 kJ mol^−1^ have been observed (Nall and Zuniga 1990). Here the rings of Y46 and Y48 pack tightly together in a typical aromatic-pair interaction, while Y67 packs against the hem group.

Applying high pressure is an elegant way to slow down ring flips and to study their activation volumes. So far, activation volumes have been determined to 27 mL mol^−1^ (Y6 in HPr) (Hattori et al. 2004), 51 mL mol^−1^ (Y35 in BPTI) and 27 mL mol^−1^ (F45 in BPTI) (Li et al. 1999). A connection with the energy of a ring flip is not known. For extremely fast ring flips, that affect order parameters, no sizeable pressure effect was observed (Kasinath et al. 2015).

Here we investigate slow ring flips in the aromatic cluster of GB1 that have been found recently (Dreydoppel et al. 2018), using ^13^C aromatic relaxation dispersion methods (Weininger et al. 2012, 2014a) in a temperature and pressure dependent way. We found that all four residues of the cluster (Y3, F30, Y45, F52) show slow ring flips. Y3, Y45 and F52 displayed nearly identical activation enthalpies and activation volumes similar to previously determined (Campbell et al. 1975; Hattori et al. 2004; Li et al. 1999; Weininger et al. 2014b), while F30 did not allow any quantification. Moreover, ring flip rates are nearly identical for Y3, Y45 (and F30) while ring flips for F52 are significantly faster. F52 is the central part of the aromatic cluster, in contact with all the other slow flipping rings. We speculate that standard activation enthalpies and faster flip rates in the center of the cluster point to correlated flip motions of F52 with all its other ring partners, each at a time.

## Materials and methods

### Protein samples

1-^13^C and 2-^13^C glucose labeled GB1 (UniProtKB P06654) was expressed and purified as described elsewhere (Lindman et al. 2006). 1-^13^C glucose labeling (Teilum et al. 2006) results in site-selective ^13^C labeled Phe and Tyr δ positions, 2-^13^C glucose labeling (Lundström et al. 2007) in site-selective ^13^C labeled Phe and Tyr ε positions. It was dissolved to a concentration of around 5 mM in 20 mM HEPES, 90% H_2_O/10% D_2_O with addition of small amounts of NaN_3_. The pH was adjusted to 7.0 in the sample.

### NMR spectroscopy

All experiments were performed at Bruker Avance III spectrometers at a static magnetic field strength of 14.1 T. Aromatic L-optimized TROSY selected ^13^C CPMG (Weininger et al. 2012) and *R*_1ρ_ (Weininger et al. 2014a) relaxation dispersion experiments have been acquired between 10 and 40 °C and 0.1 and 100 MPa. *R*_1ρ_ relaxation dispersion experiments have been recorded on-resonance. High pressure experiments were performed using a commercial 3 mm ceramic cell (Peterson and Wand 2005) (Daedalus Innovations LLC), connected to a home-built pressure generator. An aromatic ^1^H^13^C-TROSY-HSQC spectrum at − 5 °C and 200 MPa was recorded by utilizing pre-cooled air from an external device. Spectra were processed with NMRPipe (Delaglio et al. 1995) and analyzed with PINT (Ahlner et al. 2013).

### Non-averaged signals at low temperature and high pressure

At − 5 °C and 200 MPa ring flips become so slow that the individual sides of the ring could be observed in the spectra (see Table 1). This enabled us to determine the ^13^C Δ*δ* for the two sides of Y3δ (2.11 ppm), Y3ε (1.40 ppm), F30δ (5.39 ppm), F30ε (0.00 ppm) and F52ε (1.76 ppm). Previously, it was found that the shift difference Δ*δ* is not changing with temperature (Weininger et al. 2014b). Therefore, we used the derived Δ*δ* as fixed parameters in the fitting of the *R*_1ρ_ relaxation dispersion experiments, when possible. Derived ^13^C Δ*δ* might be slightly too low, because the spectrum might still be affected by exchange. However they still serve as a meaningful restraint of the fit. Furthermore, in BPTI the potential problem can be estimated to less than 1%.

**Table 1:** Effect of slow ring flips on possible positions of Phe and Tyr residues

Position	Δ*δ*^1^H (ppm)	Δ*δ*^13^C (ppm)	LB ^1^H	LB ^13^C	*R*_ex_^13^C	Ring flip	RD method
Y3δ	0.40	2.11	Yes	Yes	Yes	Slow	^1^H/^13^C
Y3ε	0.50	1.40	Yes	Yes	Yes	Slow	^1^H/^13^C
F30δ	0.84	5.39	Yes	Yes	Yes	Slow	^1^H/^13^C
F30ε	0.56	0.00	Yes	No	No	Slow	^1^H
Y33δ			No	No	No	Fast	
Y33ε			No	No	No	Fast	
Y45δ						Slow	
Y45ε			Yes	Yes	Yes	Slow	^1^H/^13^C
F52δ			No	No		Slow	
F52ε	0.00	1.76	No	Yes	Yes	Slow	^13^C

Δδ: chemical shift difference between individual signals of both sides of the ring, detected at − 5 °C and 200 MPa. LB: significant line broadening at lower temperatures. *R*_ex_: exchange contribution of *R*_2_ at lower temperatures. RD method: suitable relaxation dispersion method to study slow ring flips on this position

### Data analysis

*R*_1ρ_ relaxation dispersion data were fitted to the general equation for symmetric exchange derived by Miloushev and Palmer (2005) using fixed populations, *p*_1_ = *p*_2_ = 0.5, and treating Δδ either as a free parameter (Δδ_disp_) or fixed at the value (Δδ_spectra_) measured from HSQC spectra under slow-exchange conditions. Derived relaxation dispersion data at different temperatures and pressures were fitted simultaneously with the restrictions: *k*_flip_ (*T*_high_) > *k*_flip_ (*T*_low_), *R*_2,0_ (*T*_high_) ≤ *R*_2,0_ (*T*_low_), and *k*_flip_ (*p*_high_) < *k*_flip_ (*p*_low_).

Activation barriers of the ring flips were determined by non-linear regression of the flip rates, *k*_flip_ = *k*_ex_/2, on the temperature *T*, using the Eyring equation. The Eyring equation was parameterized as

1 $$k_{{{\text{flip}}}} = \left( {\tfrac{{k_{{\text{B}}} T}}{h}} \right) \times \exp \left[ {{{ - \left( {\Delta H^{\ddag } - T\Delta S^{\ddag } } \right)} \mathord{\left/ {\vphantom {{ - \left( {\Delta H^{\ddag } - T\Delta S^{\ddag } } \right)} {RT}}} \right. \kern-\nulldelimiterspace} {RT}}} \right]$$

where *k*_B_ and *h* are Boltzmann’s and Planck’s constants, respectively, and ∆*H*^‡^ and ∆*S*^‡^ are the activation enthalpy and activation entropy, respectively. Activation volumes ∆*V*^‡^ were determined from the pressure dependence of the flip rates according to

2 $$\left( {\frac{{\partial \ln k_{{{\text{flip}}}} }}{\partial p}} \right) = - \frac{{\Delta V^{\ddag } }}{RT}$$

Errors in the fitted parameters were estimated using Monte–Carlo simulations (Press et al. 2002); the reported errors correspond to one standard deviation.

Volume occupancies from aromatic rings in ground or transition state were estimated considering them as rotational ellipsoids with half-axes of 3.5 Å and 1.76 Å (Tsai et al. 1999; Wagner 1980). The intersection volumes of two rings in aromatic contact were then calculated using their spatial dispositions from the crystal structure (1pgb.pdb).

## Results

**Fig. 1:**
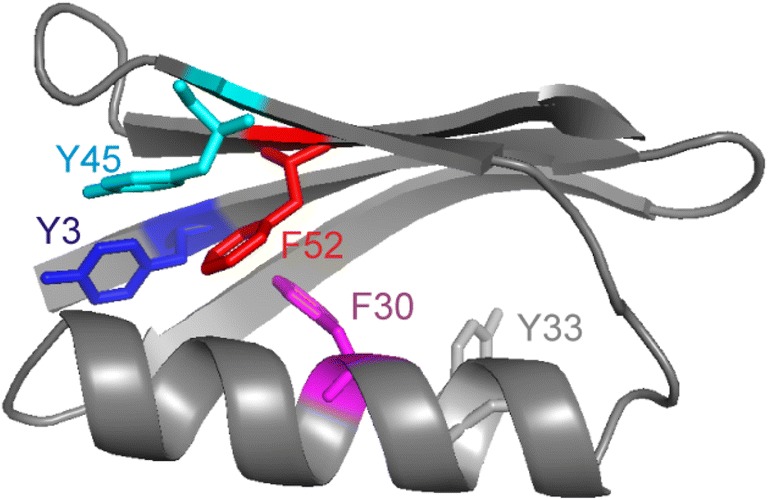
Three-dimensional structure of GB1 (1pgb.pdb) shown as ribbon presentation. Phe and Tyr side-chains are shown colored in stick representation and are labeled accordingly

Protein GB1 consists of five symmetric aromatic residues (Fig. 1), three Tyr (3, 33, 45) and two Phe (30, 52). According to their hydrophobicity, the Tyr are located closer to the surface while the Phe are buried more in the interior. The accessible surface area of the aromatic side-chains determined by GETAREA (Fraczkiewicz and Braun 1998) using 1pgb.pdb ranks the following: Y33 (70 Å^2^) ≫ Y45 (48 Å^2^) ≫ Y3 (6 Å^2^) > F30 (4 Å^2^) ~ F52 (4 Å^2^). Y33 is not involved in any particular stabilizing interactions. Y45 is stacking with the π cloud of F52 from one side, F30 is stacking with it from the other side. F52 itself is stacking with the π cloud of Y3.

### Identification of slow ring flips in GB1

**Fig. 2:**
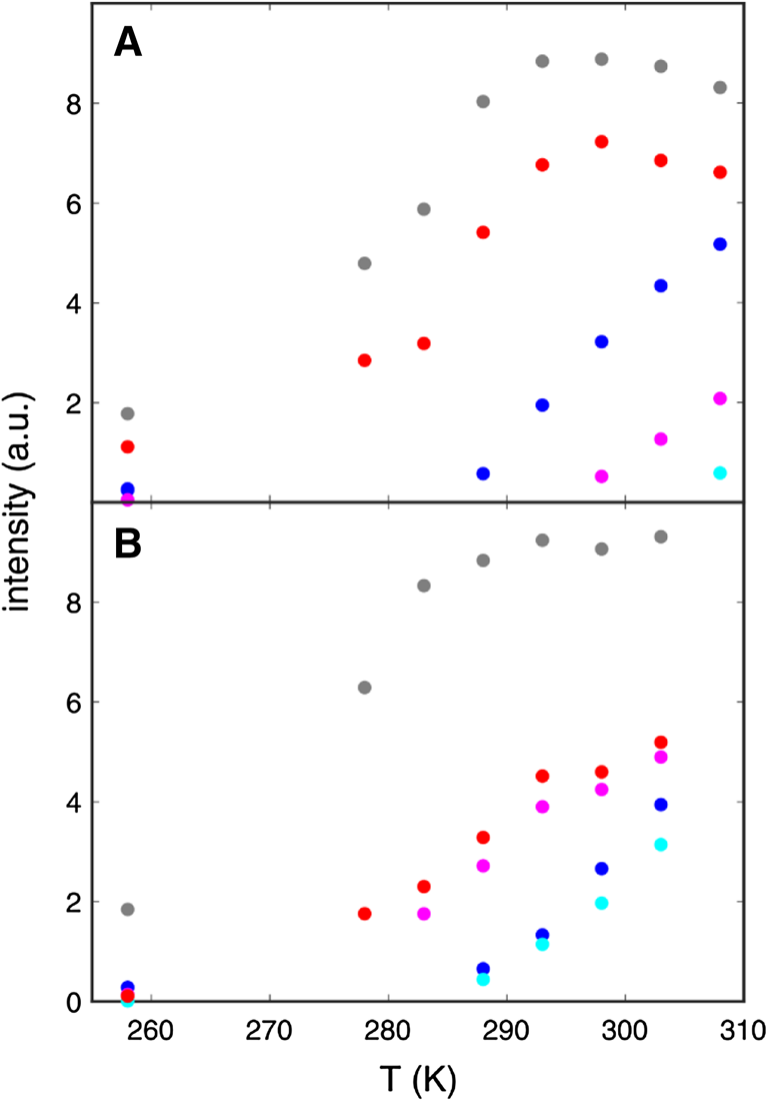
Intensity of aromatic signals that can be affected by ring flips (Phe and Tyr δ and ε). Y3 is shown in blue, F30 in magenta, Y33 in grey, Y45 in cyan and F52 in red. Normalized relative intensities of δ (**a**) and ε (**b**) are plotted against the temperature. Intensities of − 5 °C and 200 MPa are plotted at − 15 °C, since going from 0.1 to 200 MPa has roughly the same effect on the rate of ring flips than lowering the temperature by 10 K. Here the intensities of the two individual signals (δ1 and δ2, or ε1 and ε2) are the same within the symbol size. In all other cases, only averaged signals δ* and ε* (or no signals) could be observed

**Fig. 3:**
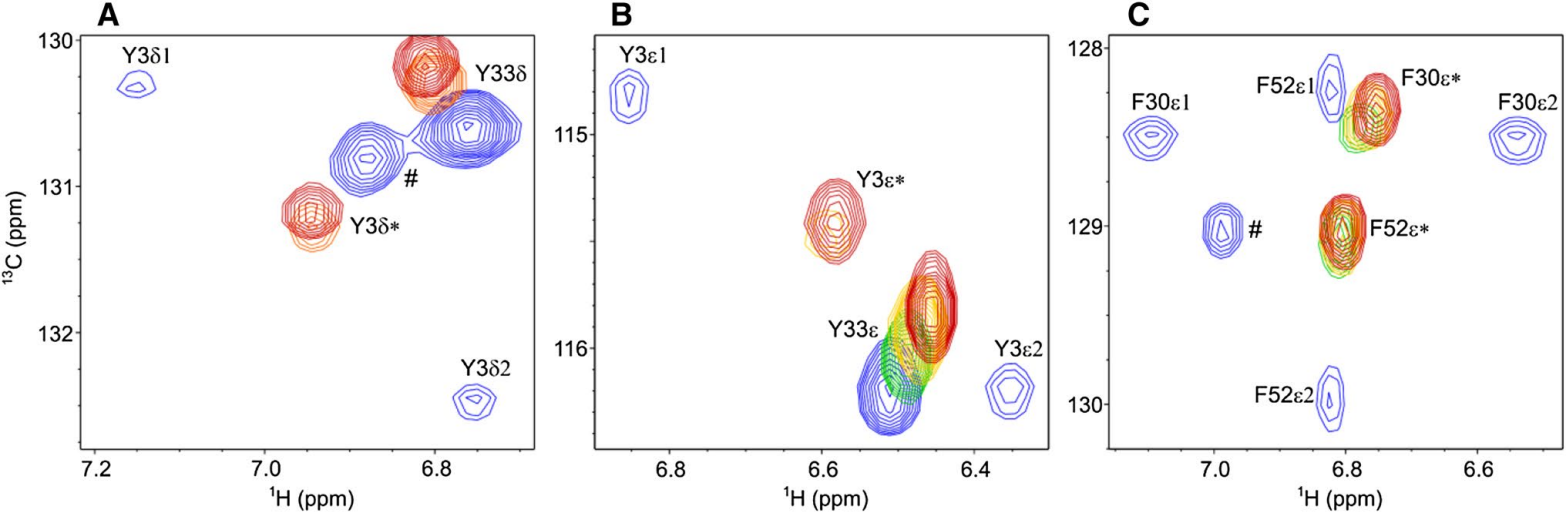
Region of **a** Tyr δ* (Y3 and Y33), **b** Tyr ε* (Y3 and Y33) and **c** Phe ε* (F30 and F52) in the aromatic ^1^H^13^C-TROSY-HSQC of GB1 at 30 °C (red), 25 °C (orange), 20 °C (yellow), 10 °C (green) and ambient pressure. The spectrum at − 5 °C and 200 MPa is shown in blue, where split signals (δ1 and δ2, or ε1 and ε2, respectively) can be observed. Signals indicated as # are caused by sample impurities which can be detected at very high S/N experiments, which were needed for the − 5 °C and 200 MPa condition, where the split signals are still severely broadened

Five averaged signals of the δ positions (δ*) and five averaged signals of the ε positions (ε*) can be observed in the aromatic ^1^H^13^C TROSY-HSQC spectra at higher temperatures. At lower temperatures signals from Y3δ/ε, F30δ/ε, Y45δ/ε and F52ε are becoming broadened (SI Fig. 1) and significantly less intense (Fig. 2). In contrast, both signals of Y33 are unaffected (other than intensity losses from slower tumbling at lower temperature). A combination of low temperature (− 5 ℃) and applied high pressure (200 MPa) is slowing down the flip processes so far, that a splitting of several signals (Y3δ, Y3ε, F30δ, F30ε and F52ε) could be observed, representing both sides of the ring in different chemical environments (Fig. 3). This effect was further elaborated by aromatic ^13^C CPMG relaxation dispersion experiments, showing an increase in ^13^C *R*_2_ (at lower temperatures) for the exact same positions where an increase in the ^13^C line width was observed (SI Figs. 1, 2). Furthermore, the kinetic process that is causing the increase in *R*_2_ is too fast to be quenched by CPMG experiments (SI Fig. 2). Taken all these findings together (Table 1), it could be established that all rings of the aromatic cluster are undergoing slow ring flips which causes an effect on ^13^C *R*_2_, line shapes and consequently signal intensity. The exception is Y33, which does not show any signs that would point towards a slow ring flip. Together with its high surface exposure we concluded that Y33 is undergoing fast ring flips. Five positions are suitable for studying slow ring flips by ^13^C relaxation dispersion methods over a range of temperature: Y3δ, Y45ε and F52ε, and to a lesser degree Y3ε and F30δ. In F30ε and F52δ ^13^C (and in case of F52 also ^1^H) is unaffected by ring flips, since the respective Δδ between both sides of the ring is (close to) zero. Y45δ is only detectable at 35 °C where ring flips are too fast to be studied by ^13^C *R*_1ρ_ experiments.

### Quantification of slow ring flips in Y3, F30, Y45 and F52 by aromatic ^13^C ***R***_1ρ_ relaxation dispersion experiments

**Fig. 4:**
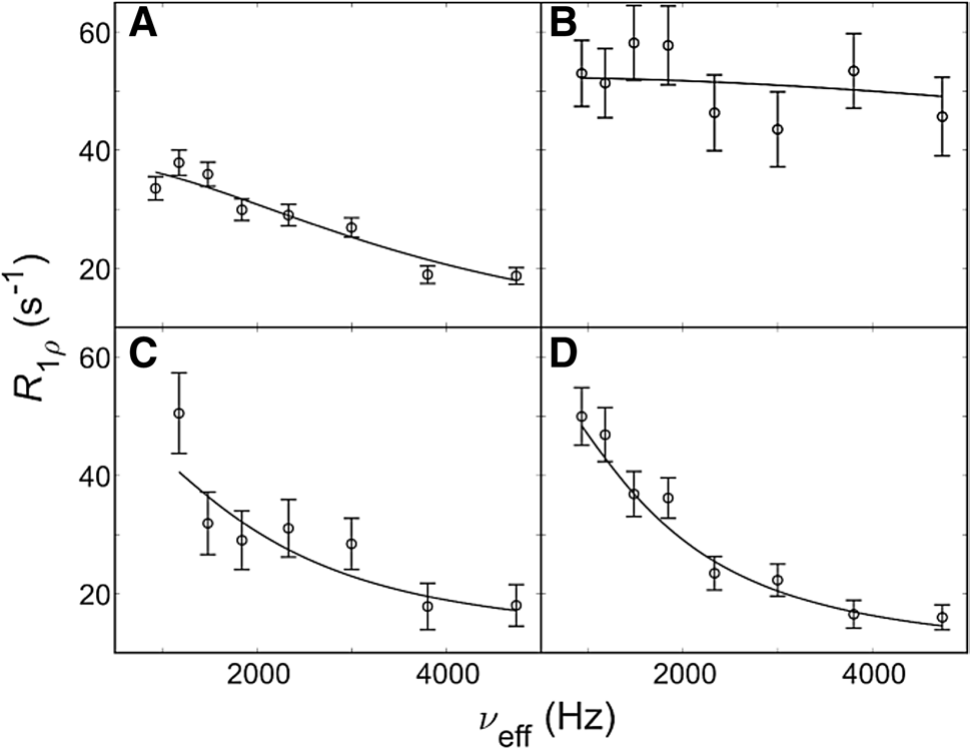
Aromatic ^13^C L-TROSY-selected *R*_1ρ_ relaxation dispersions recorded on-resonance (tilt angle *θ* > 85°) at a static magnetic field-strength of 14.1 T. Dispersion profiles for Y3δ at 25 °C (**a**), F30δ at 35 °C (**b**), Y45ε at 20 °C (**c**) and F52ε at 10 °C (**d**) are shown. Data were fitted with fixed populations *p*_1_ = *p*_2_ = 0.5 and free (Y45) or fixed chemical shift differences ∆*δ*_disp_ derived from low temperature and high pressure spectra. The resulting flip rates are: (12 ± 2) × 10^3^ s^−1^, (53 ± 4) × 10^3^ s^−1^, (6 ± 2) × 10^3^ s^−1^ and (4.8 ± 0.9) × 10^3^ s^−1^, respectively

Over the whole studied range of temperature (10 °C to 40 °C) at ambient pressure only averaged signals could be observed, or signals have been broadened beyond detection. The underlying ring flips causing the averaged signals are too fast to be captured by aromatic ^13^C CPMG relaxation dispersion experiments (Weininger et al. 2012) (SI Fig. 2), in agreement with observations on BPTI (Weininger et al. 2014b). Therefore, aromatic ^13^C *R*_1ρ_ relaxation dispersion experiments (Weininger et al. 2014a) have been applied. Relaxation dispersion profiles could be recorded for Y3δ, Y45ε and F52ε (Fig. 4a, c, d), which could be fitted to the ring flip processes. F30δ at high temperatures displays increased *R*_1ρ_ values, which cannot be quenched sufficiently (Fig. 4b). ^13^C *R*_1ρ_ relaxation dispersion experiments are allowing an accurate quantification of the ring flip processes by a simultaneous and restricted fit at different temperatures or pressures. Determined flip rates range from 4000 to 38,000 s^−1^ (75,000 s^−1^ for F30).

**Fig. 5:**
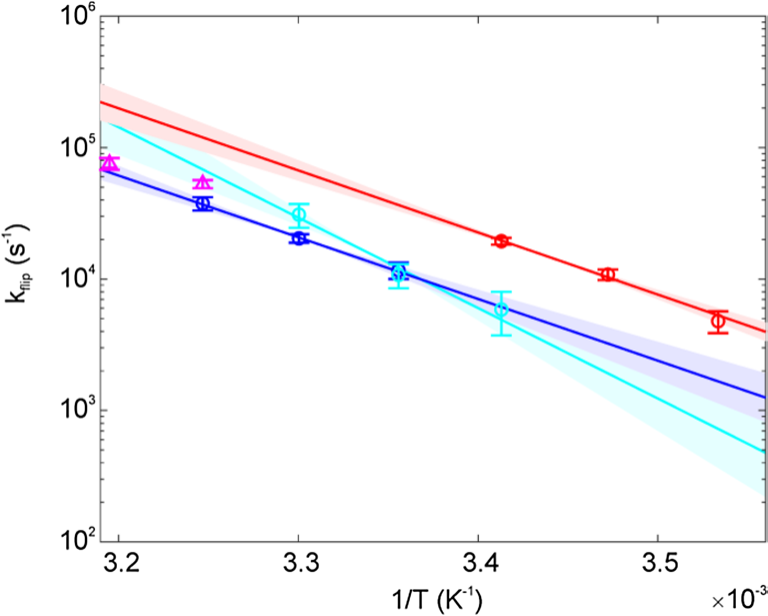
Temperature dependence of flip rates. *k*_flip_ is plotted as a function of 1/*T* for F52 (red), Y3 (blue), Y45 (cyan) and F30 (magenta). The fits are displayed as solid lines, while the uncertainties of the fits are displayed as shaded areas in the appropriate colors. The data are represented using a logarithmic y-axis to show the expected linearity, but the fit was performed using non-linear regression of *k*_flip_ on *T*

Aromatic ^13^C *R*_1ρ_ relaxation dispersion profiles could be recorded and quantified for Y3δ at 25 °C, 30 °C and 35 °C, Y45ε at 20 °C, 25 °C and 30 °C, and F52ε at 10 °C, 15 °C and 20 °C. For F30δ, only two temperatures (35 °C and 40 °C) could be used (SI Figs. 3–6). Plotting the derived flip rates against temperature (Fig. 5) reveals similar flip rates for Y3, F30 and Y45 (within margin of error), but significantly faster flip rates for F52 (at a given temperature). The latter are approximately three times higher, as can be seen from the values at 25 °C, where rates of 12 × 10^3^ s^−1^ and 11 × 10^3^ s^−1^ can be measured for Y3 and Y45, respectively, and a value of 37 × 10^3^ s^−1^ can be extrapolated for F52. Moreover, Y3 and F52, the two residues studied with the highest accuracy, display the same temperature dependence. Because of the higher flip rates, F52 had to be studied at lower temperatures. This finding is somewhat surprising, since F52 is among the most interior aromatic ring and the central part of the cluster (Fig. 1).

In order to further validate our results derived by aromatic ^13^C *R*_1ρ_ relaxation dispersion experiments, we reanalyzed the dispersion profiles for Y3δ and F52ε without the ∆*δ* fixed from information of the low temperature and high pressure spectrum. Derived ring flip rates and activation enthalpies and entropies are the same (within margin of error) with and without the additionally fixed Δ*δ * (SI Fig. 7). Furthermore, derived ∆*δ* of the fits (2.17 ± 0.20 ppm and 1.84 ± 0.09 ppm, for Y3δ and F52ε, respectively) are in excellent agreement with the ∆δ from the spectrum (2.11 ppm and 1.76 ppm).

### Y3, Y45 and F52 display similar activation enthalpies

Ring flip rates at three temperatures for Y3, Y45 and F52 could be used to derive the activation enthalpy (∆*H*^‡^) and activation entropy (∆*S*^‡^) for the individual flip processes using Eq. 1 (Fig. 5). Activation enthalpies for Y3 (87 ± 14 kJ mol^−1^) and F52 (88 ± 11 kJ mol^−1^) are virtually identical. The activation enthalpy for Y45 appears to be somewhat higher (129 ± 29 kJ mol^−1^), but could still be interpreted to be the same as for Y3 and F52, considering the significantly higher error. In fact, only the flip rate at the highest temperature for Y45, which is the least well covered in the relaxation dispersion profiles, is deviating from Y3. Activation entropies are 126 ± 46 J mol^−1^ K^−1^, 137 ± 38 J mol^−1^ K^−1^ and 275 ± 102 J mol^−1^ K^−1^, for Y3, F52 and Y45, respectively. It is not meaningful to derive activation enthalpy and entropy for F30. However, it is safe to assume that the activation enthalpy is not higher than for Y3 and F52, as indicated by the determined flip rates.

### Y3, Y45 and F52 display similar activation volumes

**Fig. 6:**
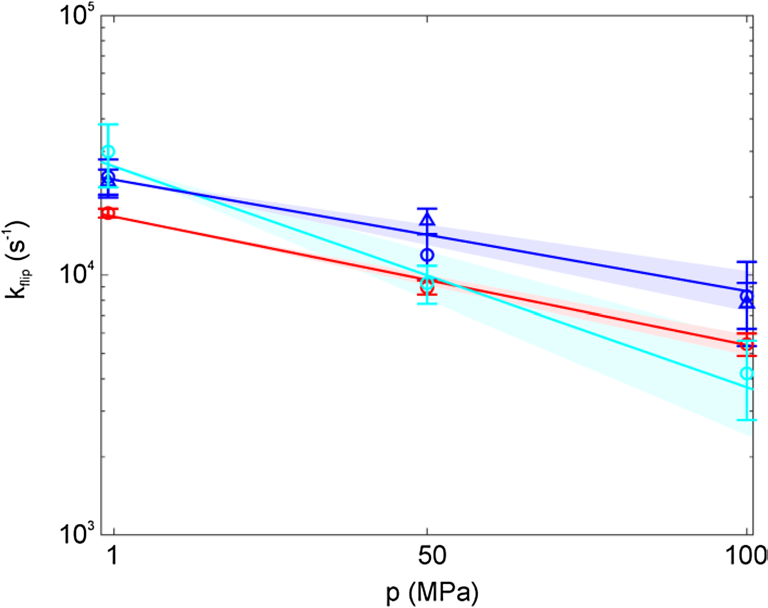
Pressure dependence of flip rates. *k*_flip_ is plotted as a function of pressure for F52 (20 °C, red), Y3 (30 °C, blue) and Y45 (30 °C, cyan). The fits are displayed as solid lines, while the uncertainties of the fits are displayed as shaded areas in the appropriate colors. The data are represented using a logarithmic y-axis to show the expected linearity, but the fit was performed using non-linear regression of *k*_flip_ on *p*

Ring flip rates for Y3, Y45 and F52 could also be recorded and quantified at three different (0.1, 50 and 100 MPa) hydrostatic pressures (SI Figs. 8–10). This allowed us to determine the activation volumes (∆*V*^‡^) of the individual ring flip processes using Eq. 2 (Fig. 6). Activation volumes for Y3 (26 ± 5 mL mol^−1^) and F52 (29 ± 2 mL mol^−1^) are virtually identical. The activation volume for Y45 appears to be somewhat higher (51 ± 11 mL mol^−1^), but could still be interpreted as the same as for Y3 and F52, considering the errors. The findings for the activation volumes thereby resemble the same general observation as for the activation enthalpies.

Again, we validated our results by an analysis without fixed ∆δ. Derived ring flip rates and activation enthalpies and entropies are again the same (within margin of error) (SI Fig. 7) and derived ∆*δ* of the fits (1.99 ± 0.31 ppm, 1.12 ± 0.09 ppm and 1.89 ± 0.07 ppm, for Y3δ, Y3ε and F52ε, respectively) are in good agreement with the ∆δ from the spectrum (2.11 ppm, 1.40 ppm and 1.76 ppm).

## Discussion

### Ring flips in the fast to intermediate NMR exchange regime

In contrast to previously reported cases of slow ring flips investigated by NMR spectroscopy (Hattori et al. 2004; Wagner et al. 1976, 1987; Weininger et al. 2014b), ring flips in GB1 do not reach the slow exchange regime, in which individual signals for both sides of symmetric aromatic rings (Phe and Tyr) could be observed, at least not at ambient pressure and temperatures above 0 °C. They are in the fast exchange regime, in which only averaged signals for both sides of symmetric aromatic rings can be observed. By lowering the temperature these signals become gradually broadened and less intense until signals are completely vanished. Since there are surprisingly very limited reports of slow ring flips in the literature, this might be the case for the vast majority of proteins. Ring flips are somewhat slow and can cause a dramatic reduction of signal intensity close to or in the intermediate exchange regime, but are not as slow to reach the slow exchange regime. Thus by more thorough temperature dependent studies of aromatic signals, many more examples of slow ring flips can be expected, despite not reaching the slow exchange regime. The aromatic ^13^C *R*_1ρ_ relaxation dispersion experiment is completely eligible to obtain correct ring flip rates (SI Fig. 7) and chemical shift differences, even without information from the slow exchange regime, and therefore allows the quantification of ring flips in the fast to intermediate NMR exchange regime. Furthermore, the determination of flip rates is robust to small variations in the chemical shift difference. In addition, high-pressure NMR is an important tool that allows additional changing of the ring flip conditions.

### Individual nuclei in aromatic side chains are affected differently

Four rings in GB1 undergo slow ring flips. In theory, ring flips could be studied on eight positions (4δ, 4ε). In practice the number of positions that can be used is significantly reduced. While some positions display differences in ^1^H and ^13^C chemical shifts and therefore can be studied by ^1^H and ^13^C methods (Y3δε, F30δ, Y45δε), others just show differences in ^1^H (F30ε) or ^13^C (F52ε), or not at all (F52δ). Similar behaviour has been observed in BPTI and rapamycin- or FK506-bound FKBP12 (SI Fig. 11) (Wagner et al. 1987; Weininger et al. 2014b; Yang et al. 2015). There are also examples of slow ring flips where both positions (δ and ε) do not display shift differences in ^1^H and ^13^C and thus are not accessible by relaxation dispersion methods (Weininger et al. 2013). Since the time scale of exchange is in the limit of *R*_1ρ_ and not CPMG relaxation dispersion experiments, and to date no ^1^H *R*_1ρ_ relaxation dispersion methods in aromatic side chains exist, F30ε is also not accessible. If the size of the chemical shift difference (for both sides of the ring) is large in ^1^H or ^13^C, the ^1^H–^13^C cross signal will be broadened over a large range of temperature, which is the case for F30δ and presumably Y45δ. Together with the upper rate limit, that can be studied by ^13^C *R*_1ρ_ relaxation dispersion experiments, and limited protein stability at higher temperatures, the final number of accessible positions is reduced even more. In case of GB1, three positions can be studied well (Y3δ, Y45ε and F52ε), while for two others the range and accuracy is less (Y3ε and F30δ). Taken all together, it requires a significant amount of screening conditions in order to conduct a quantitative study of ring flips, if the slow exchange regime cannot be reached.

### Ring flips in an aromatic cluster

The key findings for ring flips in GB1 are the following. F52, the central part of the aromatic cluster with three aromatic-aromatic contacts, is flipping at a higher rate (at a given temperature) than Y3, Y45 and F30, which flip with roughly the same rate constants (Fig. 5). The activation enthalpies for F52 (88 ± 11 kJ mol^−1^) and Y3 (87 ± 14 kJ mol^−1^) are virtually the same, no activation enthalpy could be determined for F30, but it is rather safe to conclude that it is not larger, whereas Y45 (129 ± 29 kJ mol^−1^) might also display the same activation enthalpy (within margin of error) or a slightly higher value. Activation entropies (126 ± 46 J mol^−1^ K^−1^, 137 ± 38 J mol^−1^ K^−1^ and 275 ± 102 J mol^−1^ K^−1^) are somewhat higher than previously reported ones, which range between 16 and 96 J mol^−1^ K^−1^ (Hattori et al. 2004; Weininger et al. 2014b; Yang et al. 2015). This reflects a higher loss in order in the transition state compared to the ground state of the aromatic cluster of GB1. This might be a characteristic of aromatic clusters in general and potentially is reporting a more ordered ground state. Activation volumes (Fig. 6) for F52 (29 ± 2 mL mol^−1^) and Y3 (26 ± 5 mL mol^−1^) are virtually identical; again, Y45 might display the same activation volume (within margin of error) or a slightly higher one (51 ± 11 mL mol^−1^). Previously reported activation enthalpies (86, 83, 86 and 89 kJ mol^−1^ for BPTI Y23, Y35 and F45, and HPr Y6, respectively (Hattori et al. 2004; Weininger et al. 2014b) and activation volumes (51, 28 and 27 mL mol^−1^ for BPTI Y35 and F45 (Li et al. 1999) and HPr Y6 (Hattori et al. 2004)), that have been derived on isolated aromatic rings, are very similar. The only difference is in the activation entropy.

**Fig. 7:**
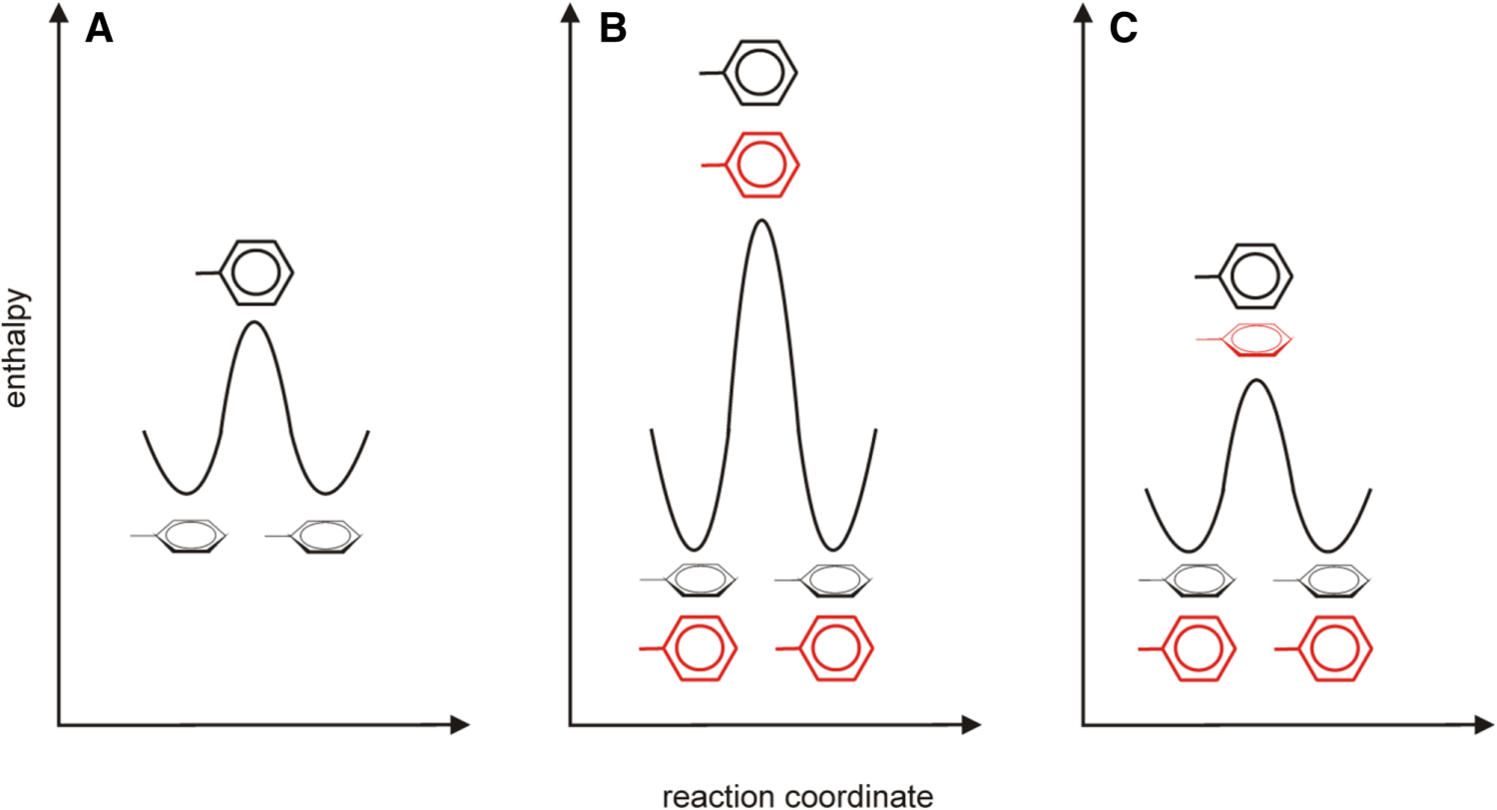
Activation enthalpy of ring flips for certain scenarios. **a** Activation enthalpy of a ring without stabilizing contacts. **b** Activation enthalpy of a ring with stabilizing contacts, in this case a stacking ring (shown in red). The stabilization of the ground state is between 5 and 10 kJ mol^−1^ (Burley and Petsko 1989). **c** Activation enthalpy of a ring with stabilizing contacts of a stacking ring (shown in red), both rings are undergoing concerted ring flips

Given all this findings, a global breathing (transient expansion) or unfolding of the aromatic cluster (which would result in higher activation enthalpies and activation volumes) can be ruled out. The ring flip process of aromatic side chains in an aromatic cluster therefore seems to be a local process, only involving a single ring or two rings in a concerted flip as will be discussed below. In fact, derived activation enthalpies and activation volumes are in very good agreement with the flipping of a single ring in an independent event. However, there are two reasons that might question this. Firstly, the central ring of the cluster which has a low accessible surface area, is flipping significantly (around three times) faster (at a given temperature). This finding is surprising, but clearly supported by the experimental data. Isolated single ring flips do not give an explanation for this. Secondly, one would have to assume that aromatic interactions (Burley and Petsko 1985, 1989) do not significantly contribute to ground state stabilization, not even for F52, the central ring with three such interactions. Aromatic stacking, however, is believed to provide between 5 and 10 kJ mol^−1^ (Burley and Petsko 1989) which would roughly translate to an increased activation enthalpy of 10 to 20 kJ mol^−1^ (Fig. 7a, b). But it might simply be, that the aromatic environment of F52 is more homogenous and better suitable for dynamic processes like ring flips and this somehow counters the enthalpic ground state stabilization by aromatic stacking.

The other possibility would be that the aromatic ring in aromatic contact with each of the others (F52) could flip in a concerted event with each one of the other rings. Under this assumption (F52 has the possibility to flip together with each Y3, F30 and Y45 in individual events) the flip rate of F52 would be the sum of all the other flip rates. In case of a concerted flip, the transition state would not be destabilized by an aromatic stacking but also stabilized, resulting in unchanged activation enthalpies (Fig. 7c). Furthermore, activation volumes could then be imagined to be reduced, because of the rings providing partial space for their partners to flip into, when rotating into the transition state. For the spatial configuration in the hydrophobic core of GB1, one obtains volume advantages of 1.6 mL mol^−1^ and 1.5 mL mol^−1^ conceded to F52 by Y45 and F30, respectively. This could partially explain why the activation volume of F52 is not significantly higher than for the others, despite being the central aromatic ring of the cluster. While all these are good reasons to speculate about concerted ring flips, it should be noted that none of the experiments performed in this study is proof for it. In order to accurately prove or disprove concerted ring flips, one has to perform MD simulations or develop challenging multiple quantum (of two rings) NMR exchange experiments (Lundström et al. 2005) through space.

## Conclusions

Here we find that the ring in the center of an aromatic cluster (F52), making aromatic stacking to three other aromatic rings, is flipping with a faster rate than the other rings, whose rates are comparable. Activation enthalpies and activation volumes in the cluster, even in its center are not increased. The only ring with a possible increase (Y45) is the ring in the cluster located most on the protein surface. We speculate that these findings are caused by correlated ring flips of F52 to at least two of its adjacent rings.

## Electronic supplementary material

Below is the link to the electronic supplementary material.

Supplementary file1 (DOCX 1502 kb)
