# Supplementary material for: Slow ring flips in aromatic cluster of GB1 studied by aromatic 13C relaxation dispersion methods
Source: J Biomol NMR. 2020 Feb 3;74(2):183–91. doi: 10.1007/s10858-020-00303-3 (PMC7080667; doi:10.1007/s10858-020-00303-3)
Supplement: Supplementary file 1 — Supplementary file1 (DOCX 1502 kb) [file 10858_2020_303_MOESM1_ESM.docx]

**Slow ring flips in aromatic cluster of GB1 studied by aromatic ^13^C relaxation dispersion methods**

Matthias Dreydoppel, Heiner N. Raum and Ulrich Weininger^*^

Institute of Physics, Biophysics, Martin-Luther-University Halle-Wittenberg, D-06120 Halle (Saale), Germany

* Correspondence:

email: ulrich.weininger@physik.uni-halle.de

phone: +49 345 55 28555

fax: +49 345 55 27161

**
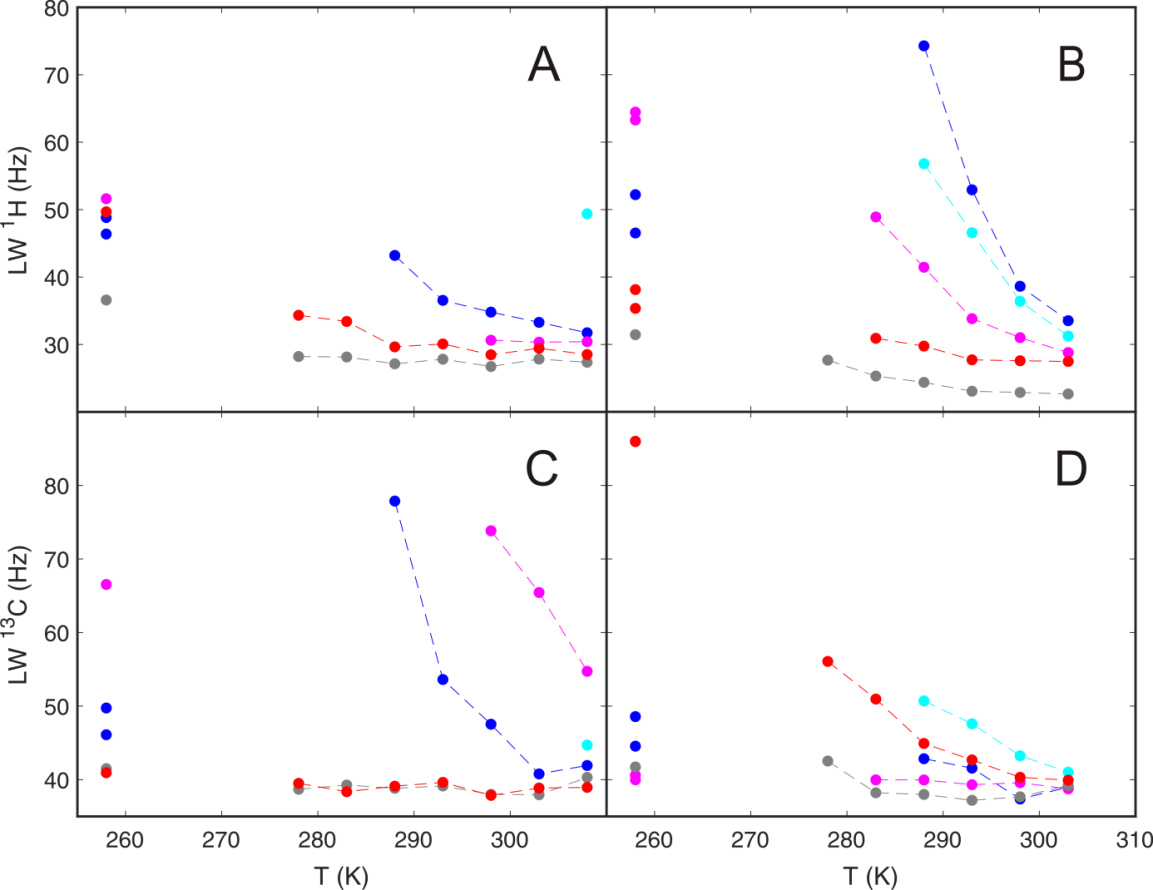
SI Fig 1:** Line widths of aromatic signals that can be affected by ring flips (Phe and Tyr  and ε). Y3 is shown in blue, F30 in magenta, Y33 in grey, Y45 in cyan and F52 in red. Absolute ^1^H (top) and ^13^C (bottom) line widths of  (AC) and ε (BD) are plotted against the temperature. Intensities of -5 °C and 200 MPa are plotted at -15 °C, analog to Fig. 2.


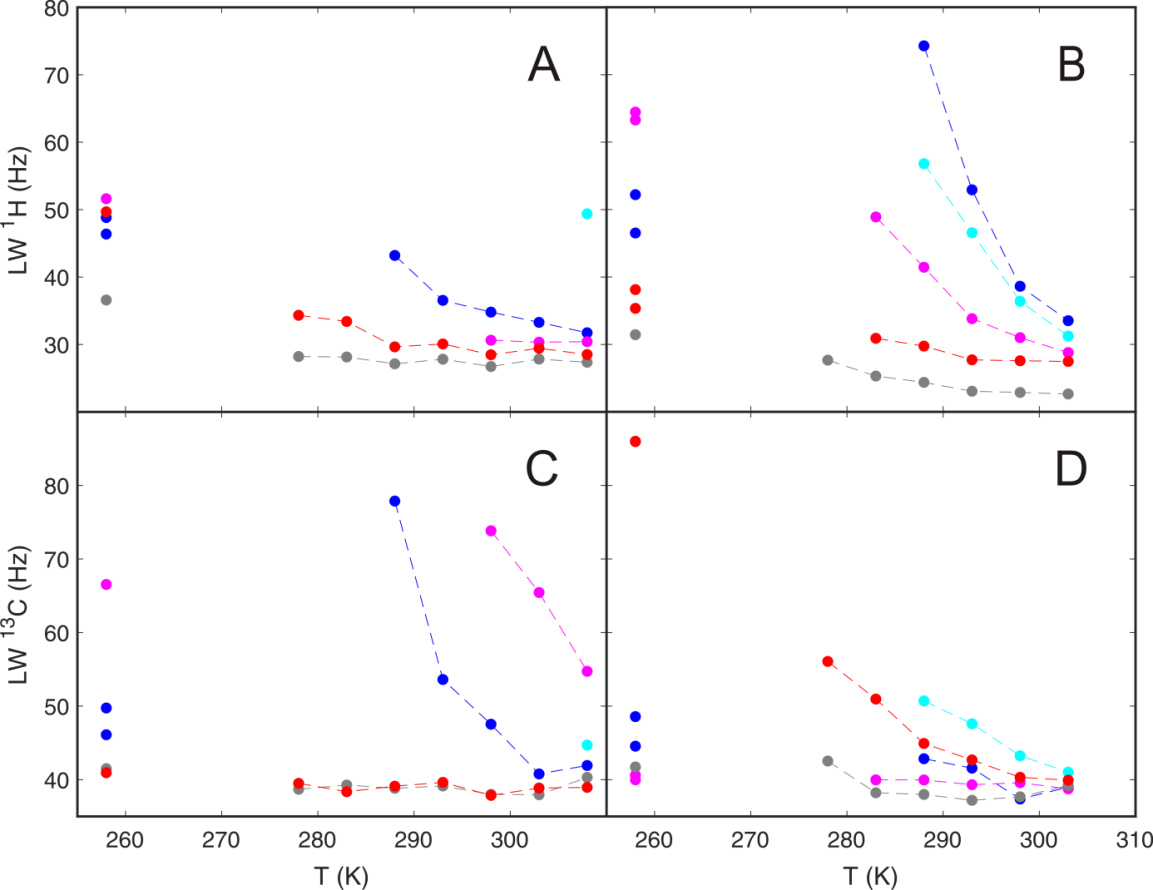


**
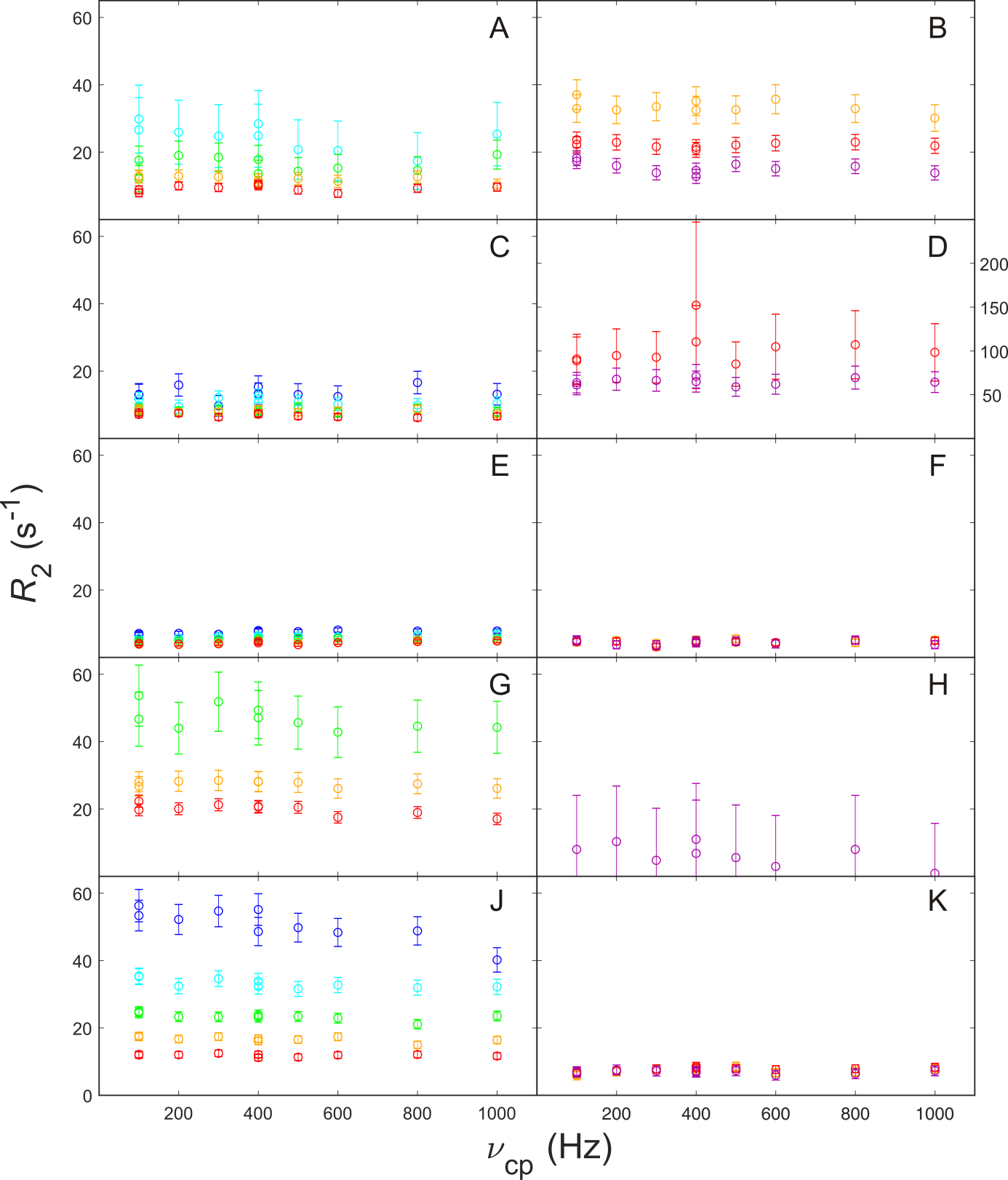
**

H

**SI Fig 2:** ^13^C aromatic CPMG relaxation dispersion profiles of Y3ε  Y3 (B), F30ε (C), F30 (D), Y33ε (E), Y33 (F), Y45ε (G), Y45 (H), F52ε (J) and F52 (K). Recorded on a 2 mM sample of GB1 at pH 7.0 and a static magnetic field strength of 14.1 T, at temperatures of 10 °C (blue), 15 °C (cyan), 20 °C (green), 25 °C (orange), 30 °C (red) and 35 °C (magenta).

**
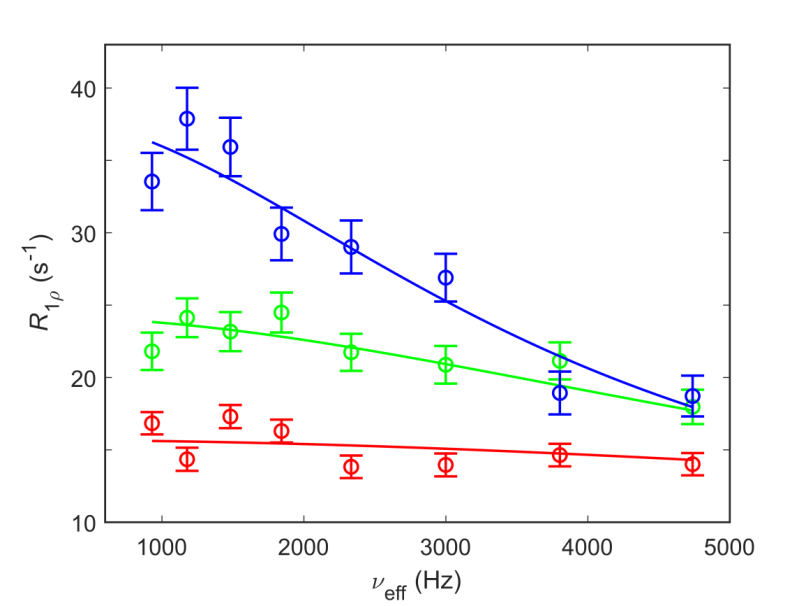
**

**SI Fig 3**: ^13^C aromatic *R*_1_*_ρ_* relaxation dispersion profiles for Y3 recorded on-resonant (*θ* > 85°) on a 2 mM GB1 at pH 7.0 and a static magnetic field strength of 14.1 T at 25 °C (blue), 30 °C (green) and 35 °C (red). The relaxation dispersions were fitted using a fixed population *p*_1_ = *p*_2_ = 0.5 and Δ*δ* fixed at the value measured from HSQC spectra under slow-exchange conditions with the restrictions: *k*_flip_ (*T*_high_) > *k*_flip_ (*T*_low_), *R*_2,0_ (*T*_high_) ≤ *R*_2,0_ (*T*_low_). Derived ring flip rate constants (*k*_flip_) are: (12 ± 2) × 10^3^ s^-1^, (20 ± 2) × 10^3^ s^-1^ and (38 ± 4) × 10^3^ s^-1^, respectively.

**
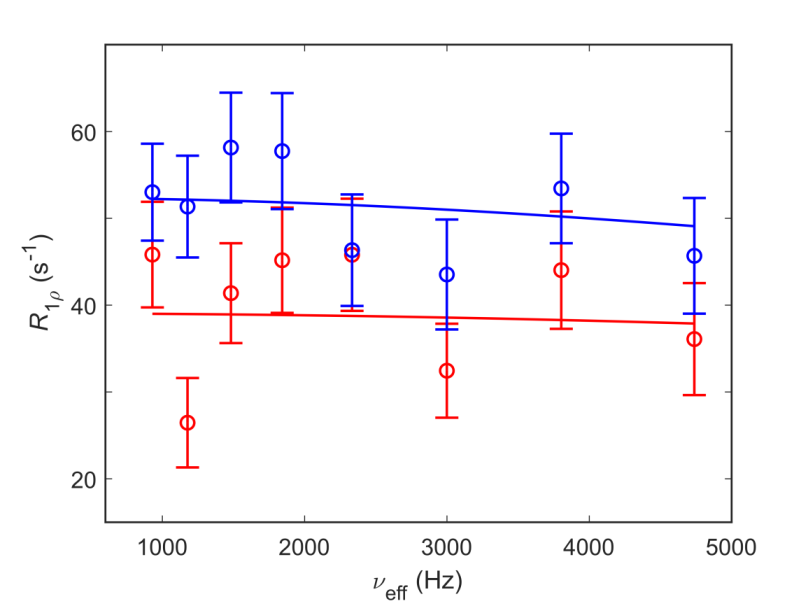
**

**SI Fig 4**: ^13^C aromatic *R*_1_*_ρ_* relaxation dispersion profiles for F30 recorded on-resonant (*θ* > 85°) on a 2 mM GB1 at pH 7.0 and a static magnetic field strength of 14.1 T at 35 °C (blue) and 40 °C (red). The relaxation dispersions were fitted using a fixed population *p*_1_ = *p*_2_ = 0.5 and Δ* * fixed at the value measured from HSQC spectra under slow-exchange conditions with the restrictions: *k*_flip_ (*T*_high_) > *k*_flip_ (*T*_low_), *R*_2,0_ (*T*_high_) ≤ *R*_2,0_ (*T*_low_). Derived ring flip rate constants (*k*_flip_) are: (53 ± 4) × 10^3^ s^-1^ and (75 ± 8) × 10^3^ s^-1^, respectively.


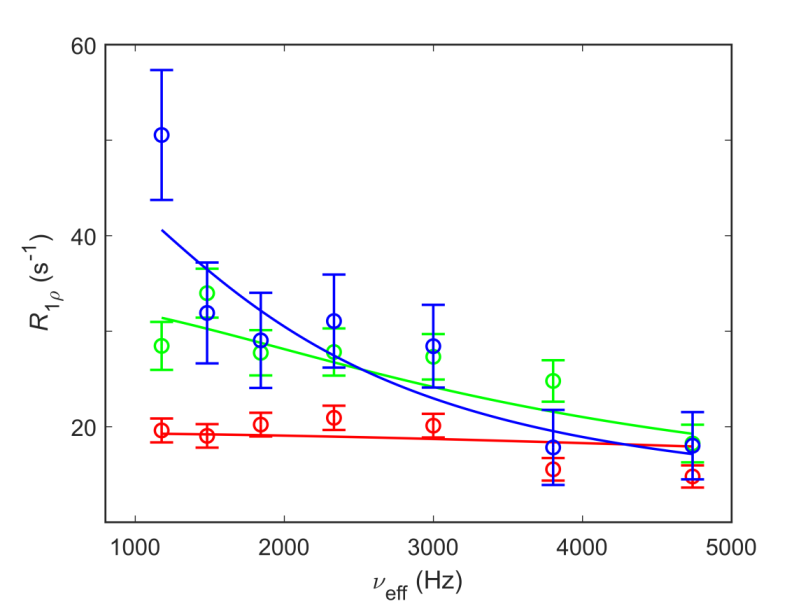


**SI Fig 5**: ^13^C aromatic *R*_1_*_ρ_* relaxation dispersion profiles for Y45ε recorded on-resonant (*θ* > 85°) on a 2 mM GB1 at pH 7.0 and a static magnetic field strength of 14.1 T at 20 °C (blue), 25 °C (green) and 30 °C (red). The relaxation dispersions were fitted using a fixed population *p*_1_ = *p*_2_ = 0.5 and Δ*δ* as a free parameter with the restrictions: *k*_flip_ (*T*_high_) > *k*_flip_ (*T*_low_), *R*_2,0_ (*T*_high_) ≤ *R*_2,0_ (*T*_low_). Derived ring flip rate constants (*k*_flip_) are: (6 ± 2) × 10^3^ s^-1^, (11 ± 2) × 10^3^ s^-1^ and (31 ± 6) × 10^3^ s^-1^, respectively.


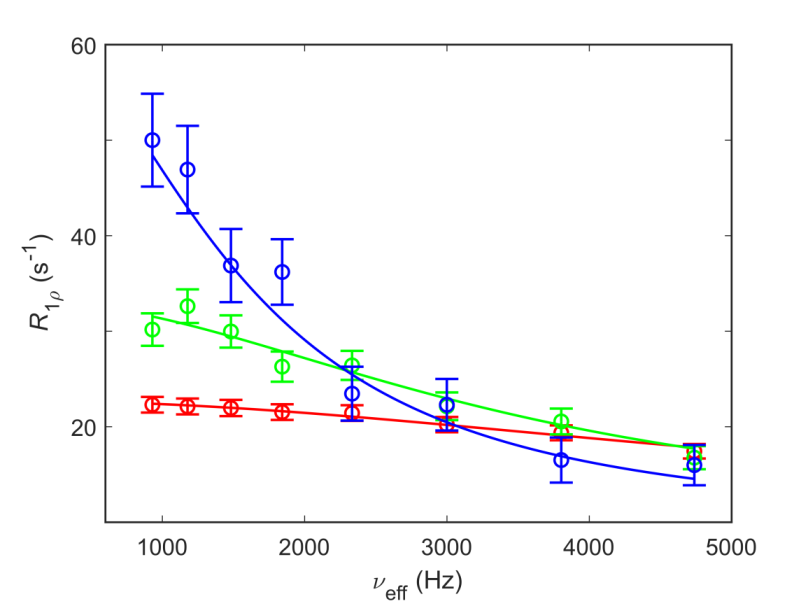


**SI Fig 6**: ^13^C aromatic *R*_1_*_ρ_* relaxation dispersion profiles for F52ε recorded on-resonant (*θ* > 85°) on a 2 mM GB1 at pH 7.0 and a static magnetic field strength of 14.1 T at 10 °C (blue), 15 °C (green) and 20 °C (red). The relaxation dispersions were fitted using a fixed population *p*_1_ = *p*_2_ = 0.5 and Δ*δ* fixed at the value measured from HSQC spectra under slow-exchange conditions with the restrictions: *k*_flip_ (*T*_high_) > *k*_flip_ (*T*_low_), *R*_2,0_ (*T*_high_) ≤ *R*_2,0_ (*T*_low_). Derived ring flip rate constants (*k*_flip_) are: (4.8 ± 0.9) × 10^3^ s^-1^, (10.8 ± 1.0) × 10^3^ s^-1^ and (19.4 ± 1.2) × 10^3^ s^-1^, respectively.

**
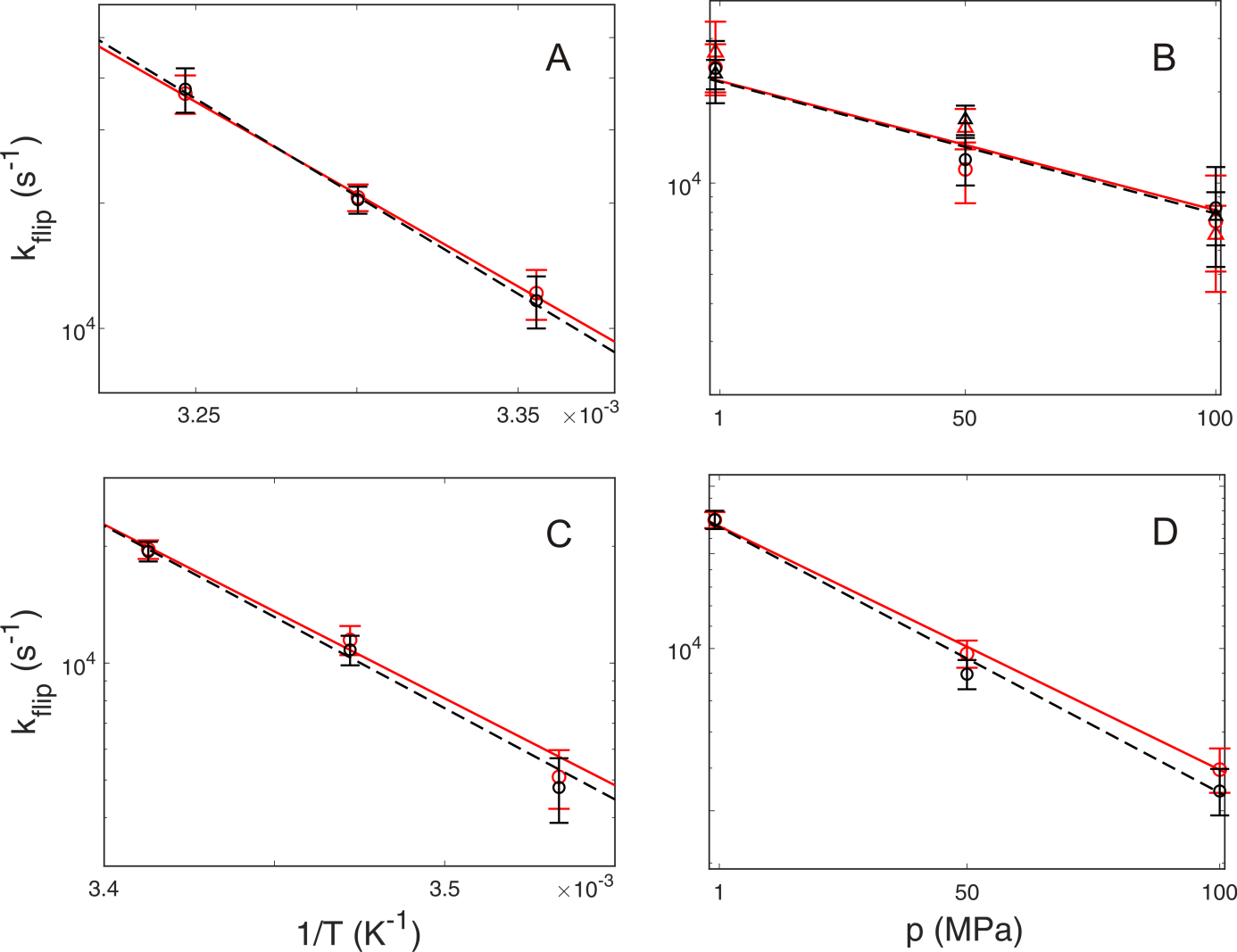
**

**SI Fig 7**: Derived flip rates by ^13^C *R*_1ρ_ relaxation dispersion experiments with (black) and without (red) fixed Δ*δ* values from the low temperature, high pressure spectra plotted against the inverse temperature (AC) or pressure (BD). A) Y3δ, B) Y3 (circles) and Y3ε (triangles), CD) F52ε. Black dashed and red solid lines represent the fits with and without fixed Δ*δ* values as function of temperature or pressure, respectively. Derived activation parameters (with / without fixed Δ*δ* ) are: Δ*H*^‡^ = (87 ± 14) / (82 ± 12) kJ mol^-1^, Δ*S*^‡^ = (126 ± 46) / (110 ± 42) J mol^-1^ K^-1^, and Δ*V*^‡^ = (26 ± 5) / (26 ± 6) mL mol^-1^ for Y3, and Δ*H*^‡^ = (88 ± 11) / (83 ± 10) kJ mol^-1^, Δ*S*^‡^ = (137 ± 38) / (122 ± 35) J mol^-1^ K^-1^, and Δ*V*^‡^ = (29 ± 2) / (27 ± 2) mL mol^-1^ for F52.

**
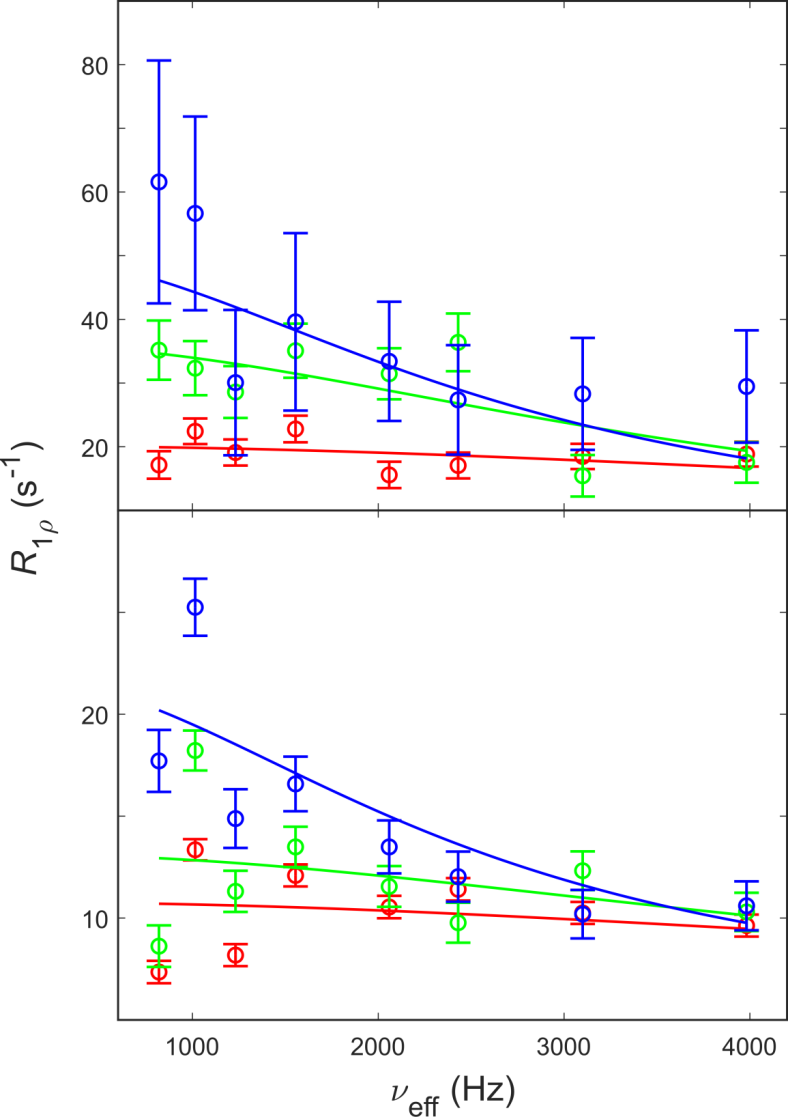
**

**SI Fig 8**: ^13^C aromatic *R*_1_*_ρ_* relaxation dispersion profiles for Y3 (top) and ε (bottom) recorded on-resonant (*θ* > 85°) on a 2 mM GB1 at pH 7.0 and a static magnetic field strength of 14.1 T at 30 °C and 0.1 (red), 50 (green) and 100 MPa (blue) hydrostatic pressure. The relaxation dispersions were fitted using a fixed population *p*_1_ = *p*_2_ = 0.5 and Δ*δ* fixed at the value measured from HSQC spectra under slow-exchange conditions with the restrictions: *k*_flip_ (*p*_high_) < *k*_flip_ (*p*_low_). Derived ring flip rate constants (*k*_flip_) are: (24 ± 4) × 10^3^ s^-1^, (12 ± 2) × 10^3^ s^-1^, (8 ± 3) × 10^3^ s^-1^ (Y3δ); and (23 ± 3) × 10^3^ s^-1^, (16 ± 2) × 10^3^ s^-1^ and (7.8 ± 1.5) × 10^3^ s^-1^ (Y3ε), respectively.

**
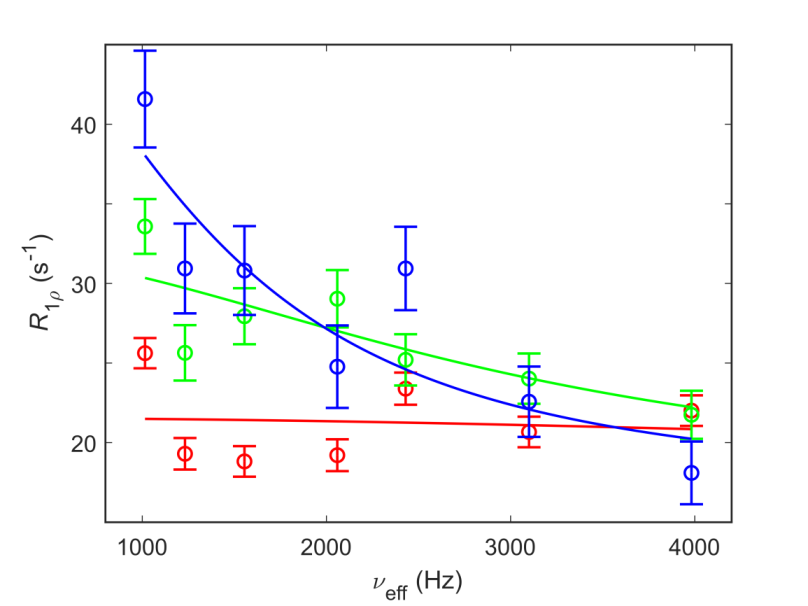
**

**SI Fig 9**: ^13^C aromatic *R*_1_*_ρ_* relaxation dispersion profiles for Y45ε recorded on-resonant (*θ* > 85°) on a 2 mM GB1 at pH 7.0 and a static magnetic field strength of 14.1 T at 30 °C and 0.1 (red), 50 (green) and 100 MPa (blue) hydrostatic pressure. The relaxation dispersions were fitted using a fixed population *p*_1_ = *p*_2_ = 0.5 and Δ*δ* as a free parameter with the restrictions: *k*_flip_ (*p*_high_) < *k*_flip_ (*p*_low_). Derived ring flip rate constants (*k*_flip_) are: (30 ± 8) × 10^3^ s^-1^, (16 ± 2) × 10^3^ s^-1^ and (4.2 ± 1.4) × 10^3^ s^-1^, respectively.

**
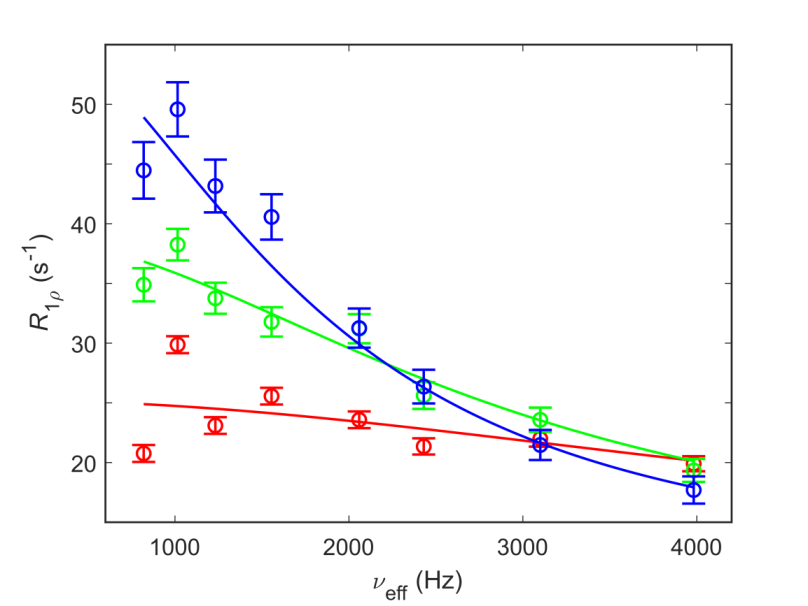
**

**SI Fig 10**: ^13^C aromatic *R*_1_*_ρ_* relaxation dispersion profiles for F52ε recorded on-resonant (*θ* > 85°) on a 2 mM GB1 at pH 7.0 and a static magnetic field strength of 14.1 T at 20 °C and 0.1 (red), 50 (green) and 100 MPa (blue) hydrostatic pressure. The relaxation dispersions were fitted using a fixed population *p*_1_ = *p*_2_ = 0.5 and Δ*δ* fixed at the value measured from HSQC spectra under slow-exchange conditions with the restrictions: *k*_flip_ (*p*_high_) < *k*_flip_ (*p*_low_). Derived ring flip rate constants (*k*_flip_) are: (17.3 ± 0.7) × 10^3^ s^-1^, (9.0 ± 0.6) × 10^3^ s^-1^ and (5.4 ± 0.5) × 10^3^ s^-1^, respectively.

**
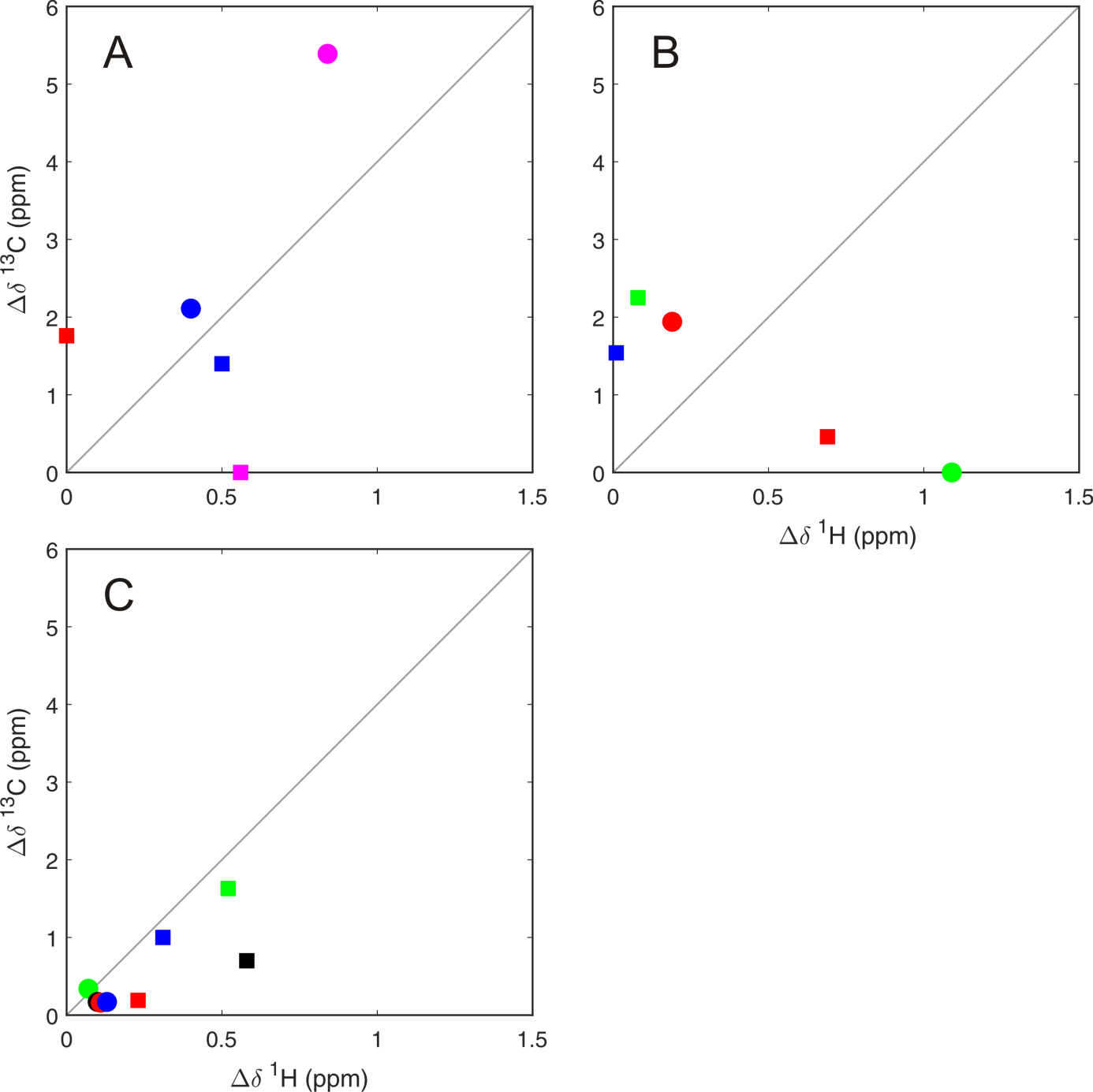
SI Fig 11**: Aromatic ^1^H and ^13^C chemical shift differences of Tyr and Phe residues,  positions indicated as circles and ε positions as squares. A) GB1 Y3, F30 and F52 in blue, magenta and red, respectively. B) BPTI Y23, Y35 and F45 in blue, green and red taken from [1 , 2]. C) FKBP12 Y26 and F99 in the rapamycin-bound state in green and red, and in the FK506-bound state in black and blue, respectively, taken from [3]. The grey lines correspond to Δ*δ* values being equivalent with respect to the ^1^H and ^13^C reference frequencies.

**References**

[1] U. Weininger, K. Modig, M. Akke, *Biochemistry* **2014**, *53*, 4519.

[2] G. Wagner, D. Bruhwiler, K. Wuthrich, *Journal of Molecular Biology* **1987**, *196*, 227.

[3] C. J. Yang, M. Takeda, T. Terauchi, J. Jee, M. Kainosho, *Biochemistry* **2015**, *54*, 6983.
